# Neuronavigated repetitive transcranial magnetic stimulation as novel mapping technique provides insights into language function in primary progressive aphasia

**DOI:** 10.1007/s11682-021-00605-6

**Published:** 2021-12-28

**Authors:** Felix Mueller-Sarnowski, Nico Sollmann, Axel Schröder, Leen Houri, Sebastian Ille, Timo Grimmer, Sandro M. Krieg, Janine Diehl-Schmid

**Affiliations:** 1 Department of Psychiatry and Psychotherapy, School of Medicine, Klinikum Rechts der Isar, Technical University of Munich, Ismaninger Str. 22, 81675 Munich, Germany; 2 Department of Medical Information Science, School of Medicine, Augsburg University, Augsburg, Germany; 3 Department of Diagnostic and Interventional Neuroradiology, School of Medicine, Klinikum Rechts der Isar, Technical University of Munich, Munich, Germany; 4 TUM-Neuroimaging Center, Klinikum Rechts der Isar, Technical University of Munich, Munich, Germany; 5 Department of Diagnostic and Interventional Radiology, University Hospital Ulm, Ulm, Germany; 6 Department of Neurosurgery, School of Medicine, Klinikum Rechts der Isar, Technical University of Munich, Munich, Germany; 7 Munich Cluster for Systems Neurology (SyNergy), Munich, Germany

**Keywords:** Navigated repetitive transcranial magnetic stimulation, Primary progressive aphasia, Language mapping, Cortical plasticity

## Abstract

Navigated repetitive transcranial magnetic stimulation (nrTMS) is an innovative technique that provides insight into language function with high accuracy in time and space. So far, nrTMS has mainly been applied in presurgical language mapping of patients with intracranial neoplasms. For the present study, nrTMS was used for language mapping in primary progressive aphasia (PPA). Seven patients (median age: 70 years, 4 males) with the non-fluent variant of PPA (nfvPPA) were included in this pilot study. Trains of nrTMS (5 Hz, 100% resting motor threshold) caused virtual lesions at 46 standardized cortical stimulation targets per hemisphere. Patients’ errors in a naming task during stimulation were counted. The majority of errors induced occurred during frontal lobe stimulation (34.3%). Timing errors and non-responses were most frequent. More errors were induced in the right hemisphere (58%) than in the left hemisphere (42%). Mapping was tolerated by all patients, however, discomfort or pain was reported for stimulation of frontal areas. The elevated right-hemispheric error rate in our study could be due to a partial shift of language function to the right hemisphere in neurodegenerative aphasia during the course of disease and therefore points to the existence of neuronal plasticity in nfvPPA. While this is an interesting finding for neurodegenerative disorders per se, its promotion might also harbor future therapeutic targets.

## Introduction

Primary progressive aphasias (PPAs) are gradually progressive, neurodegenerative disorders predominantly affecting speech and language. The non-fluent variant of PPA (nfvPPA) is characterized by a predominant posterior fronto-insular atrophy of the language-dominant hemisphere. Patients present with a non-fluent, effortful, halting speech that is dominated by obvious word-finding difficulties and agrammatism. Language production is simplified, phonematic paraphasias are frequent. Patients often present with apraxia of speech while dysarthria occurs less frequently (Gorno-Tempini et al., 2011).

Therapeutic options are scarce and consist mainly of speech therapeutic approaches. A few studies have made first, not very successful attempts to improve language function using repetitive transcranial magnetic stimulation (rTMS) and transcranial direct current stimulation (tDCS) (Norise & Hamilton, 2017; Cotelli et al., 2012, 2020). However, targeted regions and stimulation parameters vary widely in these attempts, including anodal tDCS targeting of the left inferior frontal gyrus, high-frequency rTMS of the whole left hemisphere, high-frequency rTMS of the left prefrontal cortex, or anodal (facilitative) stimulation of the left posterior peri-Sylvian region. This variability is the consequence of the sparse rationale for such interventions.

Knowledge about language function and compensatory mechanisms in the individual patient is a prerequisite for a more personalized and thus more efficient neurostimulation in nfvPPA. Positron emission tomography (PET) and functional magnetic resonance imaging (MRI) during a language task could generate such information, but only with limited resolution in time. Navigated rTMS (nrTMS) has been shown to be a reliable technique in order to perform presurgical language mapping in patients with intracranial neoplasms (Krieg et al., 2014; Picht et al., 2013; Sollmann et al., 2017b; Tarapore et al., 2013). Therefore, the primary aim of our pilot study was to investigate, which brain regions are involved in tasks of language and speech in patients with nfvPPA using nrTMS. Secondly, we wanted to investigate, how patients with nfvPPA tolerate nrTMS.

## Methods

### Patients

Patients with nfvPPA according to the Gorno-Tempini criteria (Gorno-Tempini et al., 2011) were enrolled from our outpatient clinic for cognitive disorders. Patient characterization comprised neurologic and psychiatric examination, and extensive neuropsychological testing. (Table 1).

**Table.1:** Patient characteristics

Patient		#1	#2	#3	#4	#5	#6	#7
sex		male	female	male	male	female	male	female
age		73	70	56	68	75	55	74
age at onset		70	66	54	67	72	52	71
education (years)		15	12	15	20	18	19	12
handedness*		right	left mixed (forced right)	left (forced right)	right	right, mixed	right	right
psychotropic drugs		-	mirtazapine 7.5 mg	-	escitalopram 5 mg quetiapine 100 mg	citalopram 20 mg	amitriptyline 5 mg	-
FTLD-CDR	sum of boxes	3	1	0.5	3.5	2	5	5.5
	language	0.5	0.5	0.5	0.5	0.5	2	2
semantic fluency (animals)		7 (-2.8 z)	9 (- 2.4 z)	16 (-1.6 z)	18 (-1.2 z)	15 (-2.2 z)	2 (-4.5 z)	6 (-2.9 z)
phonematic fluency (S-words)		4 (-2.2 z)	7 (-1. z)	10 (-1.0 z)	5 (-2.6 z)	7 (-1.9 z)	3 (-3.3 z)	1 (-3.3 z)
15 item BNT (/15)		14 (-0.4 z)	15 (1.3 z)	15 (0.5 z)	15 (0.5 z)	15 (1.0 z)	4 (-5.9 z)	5 (-4.4 z)
MMSE (/30)		24 (-3.1 z)	29 (-0.2 z)	29 (-0.8 z)	29 (-0.7 z)	29 (- 0.6 z)	20 (-5.7 z)	17 (-6.0 z)
CERAD word list learning (/30)		12 (-2.3 z)	21 (-0.3 z)	24 (0.6 z)	24 (0.9 z)	19 (-1.2 z)	15 (-2.2 z)	16 (-1.8 z)
CERAD word list delayed recall (/10)		4 (-1.2 z)	8 (0.1 z)	8 (0 z)	7 (-0.4 z)	7 (-0.8 z)	2 (-3.0 z)	5 (-1.3 z)
CERAD figure copy (/11)		7 (-2.9 z)	9 (-1.3 z)	11 (0.5 z)	10 (-1.4 z)	11 (0.6 z)	11 (0.3 z)	10 (0.5 z)
CERAD figure recall (/11)		4 (-2.5 z)	7 (-0.9 z)	11 (0.8 z)	9 (-1.0 z)	11 (1.0 z)	11 (0.7 z)	5 (-1.5 z)
trail making A (seconds)		> 180	82 (-1.8 z)	46 (-0.9 z)	70 (-1.8 z)	65 (-1.2 z)	45 (-0.7 z)	43 (0.3 z)
trail making B (seconds)		> 300 (-2.5 z)	> 300 (- 2.5 z)	138 (-1.7 z)	283 (-3.3 z)	194 (-2.2 z)	180 (-2.6 z)	121 (-0.2 z)
AAT token test		5	12	12	1	3	42	5
AAT token test age adjusted		0	6	9	0	0	38	3
AAT loud reading		29	26	26	30	28	27	29
AAT composing words		28	28	29	30	30	29	24
AAT writing to dictation		28	29	24	30	30	27	8

CERAD = Consortium to Establish a Registry in Alzheimer’s Disease - neuropsychological battery, BNT = Boston Naming Test, FTLD-CDR = FTLD specific clinical dementia rating; MMSE = Mini-Mental-State Exam, WMS = Wechsler Memory Scale, AAT Aachen Aphasia Test; * handedness was assessed with the Edinburgh Handedness Inventory (Oldfield, 1971)

Patients were examined with the German Version of the Consortium to Establish a Registry in Alzheimer’s Disease (CERAD) neuropsychological battery (Morris, 1993), which includes the Mini-Mental-State Examination (Folstein et al., 1975) and parts of the Aachener Aphasie test (Huber et al., 1983) (Table 1). Dementia severity was rated using the frontotemporal lobar degeneration-specific clinical dementia rating (FTLD-CDR) (Knopman et al., 2008). Neuropsychological assessment was performed within a maximum of eight weeks (median 13 days) before or after the nrTMS mappings.

In all patients cranial MRI was performed at 3 Tesla (Ingenia Elition X, Philips Healthcare, Best, The Netherlands). The protocol included a three-dimensional (3D) T1-weighted gradient echo sequence (repetition time/echo time: 9/4 ms, 1 mm^3^ isovoxel covering the whole head, no application of an intravenous contrast agent) for neuronavigation. Furthermore, ^18^ F-FDG-PET was performed in all patients.

### Navigated rTMS

#### Examination of naming performance

Naming task parameters were adapted to each individual participant’s performance prior to the nrTMS mappings. During two baseline sessions the patients had to name schematic black and white drawings of 80 common objects (one or two syllables), based on the Snodgrass and Vanderwart images (Snodgrass & Vanderwart, 1980). The objects were presented on a screen one after another. After each of two naming sessions without stimulation (baseline sessions), the object was sorted out when the patient was not able to name the object promptly and correctly.

**Fig. 1:**
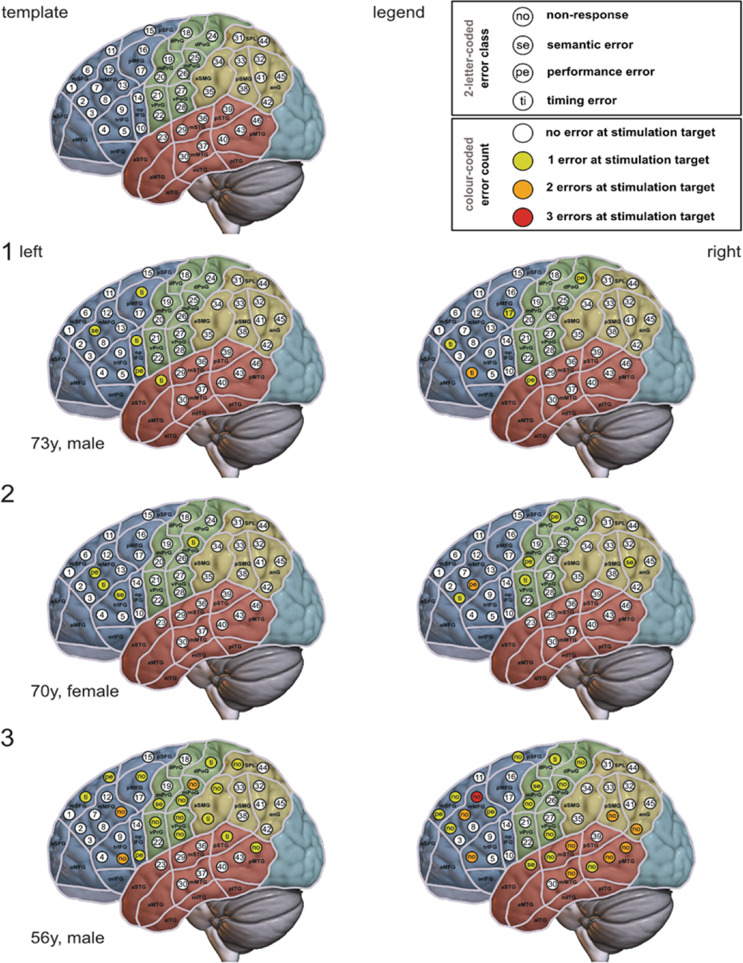

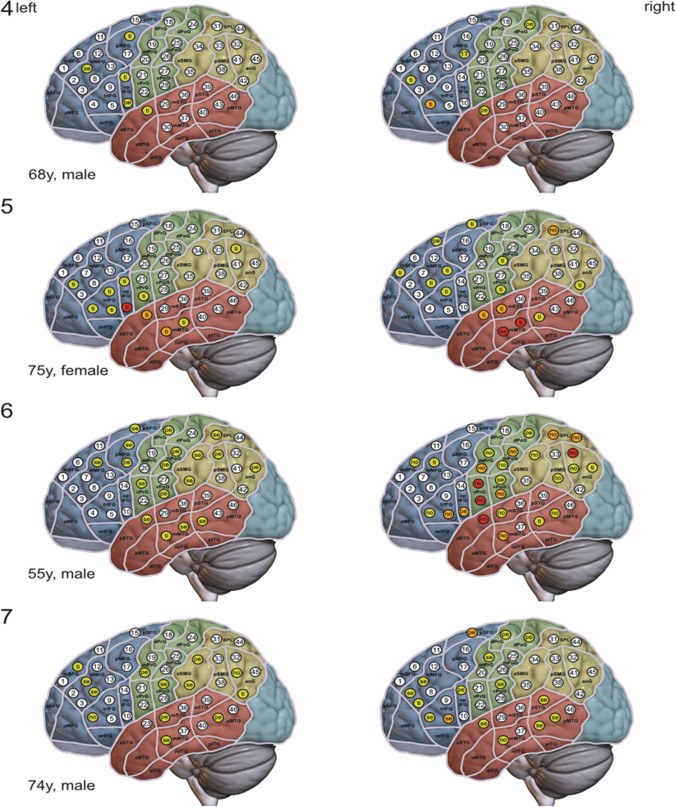
Language maps – For all patients (1-7) nrTMS language-mappings of the left hemisphere (left column) and right hemisphere (right column) are shown. At each of the 46 standardized stimulation targets in every hemisphere a color code indicates the error count (white = no error, yellow = 1 error, orange = 2 errors, red = 3 errors) while a 2-letter code discloses the error class ranking highest among the errors at the target. A slightly modified parcellation scheme according to Corina et al. is displayed on top of the MNI 2009b asymmetric template. Regions belonging to one lobe share the same color: blue in the frontal lobe, green in the central lobe, brown in the temporal lobe, ocher in the parietal lobe, and turquoise in the occipital lobe The right hemisphere is projected into a template of the left hemisphere in order to facilitate comparability

Two patients (#6 and #7) were not able to correctly name the majority of objects (errors in 84.4%, and 59.7%, respectively). Consequently, the nrTMS examination based on object naming seemed not feasible. However, both patients showed less difficulties in a number naming task (patient #6: 24% errors; patient #7: 36.1% errors). Therefore, the object naming task was replaced by a number (Figs. 0, 1, 2, 3, 4, 5, 6, 7, 8 and 9) naming task in these two cases.

#### Language mapping

Language mapping by nrTMS was performed with the Nexstim eXimia 5.1 NBS system (Nexstim Plc., Helsinki, Finland) using a figure-of-8 coil. The technical procedure and the protocol for language mapping has been described in detail previously (Picht et al., 2013; Tarapore et al., 2013; Krieg et al., 2014; Ille et al., 2016; Sollmann et al., 2017a, b; Krieg et al., 2013). In brief, cortical tissue was temporarily inhibited by a train of five repetitive nrTMS pulses at standardized stimulation targets in each hemisphere during a naming task. For neuronavigation, a co-registration of each patient’s head and the according 3D MRI data was performed. Stimulation intensity was calibrated by the resting motor threshold (rMT) of hand muscles. The rMT is defined as lowest TMS intensity provoking a 50 µV peak-to-peak amplitude in the relaxed right first dorsal interosseus muscle in five out of ten stimulations (Rossini et al., 2015).

Forty-six standardized stimulation targets per hemisphere, distributed over 21 gyral segments, were set up on each patient’s reconstructed head model within the nrTMS software according to a template based on a parcellation scheme described by Corina and colleagues (Corina et al., 2005; Tussis et al., 2016, 2017). The pre-selected objects (or numbers) were presented time-locked to nrTMS trains (5 Hz, 100% rMT). Keeping the coil perpendicular to the gyrus three nrTMS trains of five stimuli were applied at each target simultaneously with the picture presented on the screen. Thus, each of the targets was stimulated three times during the presentation of three pictures, leading to 276 stimulations per exam in each patient. The patients had to name the objects (or numbers) as quickly as possible. Then the stimulating coil was randomly moved to the next target during the inter-picture interval of 2.5 s, and the picture-to-trigger-interval was 0 ms with a display time of 700 ms for each picture (Krieg et al., 2014, 2016, 2017; Sollmann et al., 2014, 2017b; Tussis et al., 2016, 2017, 2020).

#### Classification of naming errors

In accordance with prior nTMS studies errors were classified into „no response“ (no, strongly distorted or incomplete utterance), „semantic error“ (incorrect choice of word), „performance error“ (correct choice of word, but utterance or assembly incorrect), and “timing error” (delay, hesitation) (Krieg et al., 2016; Lioumis et al., 2012; Picht et al., 2013; Sollmann et al., 2014, 2017b). The error classes “neologism” and “circumlocution” originally described as error classes by Corina and colleagues, were summarized as semantic errors. Phonological errors were subsumed among performance errors.

#### Analysis

For the detailed and standardized assessment – blinded to the sites of cortical stimulation – the baseline examination of the naming performance and the nrTMS examination with patients’ answers were video-recorded (Picht et al., 2013; Sollmann et al., 2014). The videos recorded during nrTMS language mappings were analyzed, and any naming impairment was compared with the corresponding baseline response. Videos were rated in ELAN, an annotation tool for multi-media resources developed by the Max Planck Institute for Psycholinguistics that integrates tightly with Praat (version 6.1.53; Praat: doing phonetics by computer, http://www.praat.org/) (Boersma & van Heuven, 2001; Wittenburg et al., 2006). The spatial distribution of the naming errors that resulted from the virtual lesions revealed a map of cortical language areas. Additionally, the calculation of error rates was performed for each subject and each hemisphere by dividing the number of errors by the number of stimulations (per stimulated spot). Naming errors presumably due to pain or discomfort related to the stimulation were discarded.

## Results

Seven patients with nfvPPA participated in the study. Patient characteristics and neuropsychological test results are displayed in Table 1. Clinical Gorno-Tempini criteria for nfvPPA were fulfilled in all patients. All participants showed a frontal and temporal atrophy in cranial MRI and a left frontal hypometabolism in ^18^ F-FDG-PET. In five patients an amyloid PET was obtained that was negative in all cases. In the remaining two patients a lumbar puncture was performed: CSF levels of amyloid-β42 were normal.

### Error counts

As displayed in Table 2 a total of 353 naming errors during 1932 stimulations was observed in nfvPPA patients, which amounts to an error rate of 18.2%. The majority of errors occurred during stimulation of the frontal lobes (34.3%), followed by the temporal lobes (24.6%) and the central lobes (22.1%).

**Table.2:** Errors per lobe and hemisphere during object naming (patient 1 – 5) and number naming (patient 6, 7)

Patient	Hemisphere	Frontal	Central	Temporal	Parietal	Sum hemisphere	Sum brain
1	left	31	12	14	17	74	144
	right	20	15	18	17	70	
2	left	3	1	0	0	4	11
	right	3	3	0	1	7	
3	left	7	8	2	3	20	55
	right	12	6	13	4	35	
4	left	4	0	1	0	5	11
	right	4	1	1	0	6	
5	left	7	1	5	1	14	37
	right	6	2	12	3	23	
6	left	4	6	4	5	19	64
	right	7	17	9	12	45	
7	left	4	2	3	4	13	31
	right	9	4	5	0	18	
	Sum left Sum right					149 (42%) 204 (58%)	
	Sum total	121(34.3%)	78(22.1%)	87(24.6%)	67(19.0%)	353 (100%)	

Error counts per hemisphere and lobe summed up to the overall error count in the last column

Patients #2 an #3 are forced right-handed

Error classes and distribution of errors in both hemispheres are shown in Table 3; Fig. 1. Overall, more errors were induced in the right hemisphere (204/353 errors, 58%) than in the left hemisphere (149/353 errors, 42%). Timing errors and non-responses were most frequent (39% and 35% of all errors).

**Table.3:** Error classes per hemisphere

Error class	Errors left hemisphere	Errors right hemisphere	Total errors
no response	49 (32.9%)	76 (37.2%)	125 (35.4%)
semantic	21 (14.1%)	24 (11.8%)	45 (12.8%)
performance	18 (12.1%)	28 (13.7%)	46 (13.0%)
timing	61 (40.9%)	76 (37.3%)	137 (38.8%)
total	149 (100%)	204 (100%)	353 (100%)

### Tolerability

Overall, the nrTMS examination was tolerated well by six out of seven patients. All of the patients stated after the examination that they would be willing to undergo further nrTMS sessions. However, the majority of patients disclosed considerable discomfort and pain (Table 4). As observed in the video recordings and confirmed by the participants, pain and discomfort were limited to stimulation of frontal regions.

**Table.4:** Discomfort and pain – patient estimation on a 10-point Likert scale (0= no discomfort/ pain)

Patient	#1	#2	#3	#4	#5	#6	#7
pain left hemisphere	5	7	2	0	5	1	0
pain right hemisphere	6	5	3	0	5	1	8
discomfort left hemisphere	6	4	2	0	5	0	0
discomfort right hemisphere	7	3	3	0	5	0	0

## Discussion

To our knowledge the present study is the first that performed language mapping in neurodegenerative aphasia using nrTMS. Specifically, nrTMS induced an overall error rate of 18% in nfvPPA patients. Previous studies using nrTMS in healthy individuals have described similar error rates (Krieg et al., 2016; Sollmann et al., 2014, 2017b). Timing errors and no responses were most frequent in nfvPPA patients (39% and 35% of all errors), in line with the fact that halting speech is one of the two core features of nvfPPA (Gorno-Tempini et al., 2011).

Although we observed an obvious inter-individual variability regarding error rates and language maps, stimulation of the temporal, central, and parietal lobes induced fewer errors than stimulation of frontal areas. This goes along with the fact that nfvPPA is characterized by imaging abnormalities in the posterior fronto-insular region (Gorno-Tempini et al., 2011). Specifically, the middle middle frontal gyrus (mMFG) was identified as a region with prevalent errors in both hemispheres, which seems to extend to the posterior MFG (pMFG) in several cases. Cortical thickness in the MFG of both hemispheres was shown to correlate with reduced fluency in PPA (Rogalski et al., 2011). Furthermore, the MFG is also a spot of nfvPPA-typical hypometabolism in FDG-PET (Routier et al., 2018). In a recent tDCS treatment trial, individuals with reduced left MFG volumes benefited more from tDSC than those receiving sham (de Aguiar et al., 2020). Other regions with prevalent errors are the triangular inferior frontal gyrus (trIFG) and opercular IFG (opIFG), which mostly overlap with Broca’s area in the left hemisphere. While this replicates the importance of Broca’s area for human language in general, language function in nfvPPA is by no means confined to these regions, but widespread. In five out of seven patients (all except patients #4 and #5) nTMS language maps point towards a particular role of the central gyri in nfvPPA language production. This is consistent with findings in stroke patients who showed involvement of the precentral gyrus in apraxia of speech (Itabashi et al., 2016).

Surprisingly, more errors were induced in the right hemisphere (58%) than in the left hemisphere (42%). A shift of language function towards the right or unaffected hemispheres has already been suggested in stroke, epilepsy, and intracranial neoplasms (Anglade et al., 2014; Baciu & Perrone-Bertolotti, 2015; Ille et al., 2016; Krieg et al., 2013). There is broad consensus that this phenomenon indicates cortical reorganization. Despite some evidence from functional imaging for a language shift in nfvPPA patients (Vandenbulcke et al., 2005), the general understanding so far is that patients with nfvPPA subtypes are not remapping language functions to contralateral networks. They appear to use pre-existing language areas, but with progressively declining efficiency (Norise & Hamilton, 2017). Notably, these assumptions are based on comparatively weak evidence (Wilson et al., 2010). Yet, cortical reorganization in nfvPPA and other neurodegenerative disorders fuels hope that nrTMS might become a treatment option, either by stimulation of compensatory right hemispheric networks or by stimulation of pathologically weakened left hemispheric networks in order to enhance the remaining language function (Norise & Hamilton, 2017).

However, an alternative explanation for the distributions of error rates in each hemisphere could also directly relate to degenerative processes: a relative increase in the number of errors following stimulation could depend on the high degree of impairment of neural plasticity in the left hemisphere, which is the main focus of the neurodegenerative process in PPA. Further studies that combine nrTMS with functional or metabolic imaging to specifically stimulate circumscribed areas with altered activity profiles may help to shed light on the distinct causes for the observed error distributions between hemispheres.

The present study has some obvious limitations. First, the sample size is small and the patient sample was not homogenous regarding handedness. Second, not all patients were able to perform an object naming task so that a number naming task was used in two patients. Third, a general obstacle of language mapping with nrTMS is that stimulation targets at the edge of the frontal lobe are not included since stimulation at these targets causes intolerable discomfort in many patients. Consequently, the polar to orbital parts of the frontal lobe are not covered. In addition, since naming errors that were caused by discomfort or pain were discarded from the analysis, the error rate in frontal regions was potentially underestimated. Finally, the study lacks healthy control probands that would allow a comparison of the performance.

However, the present study is the first to show that language mapping in neurodegenerative aphasia using nrTMS is feasible and offers unique insights into language function by combining good temporary with good spatial resolution. Evaluating the individual language function might be a prerequisite for any targeted, personalized stimulation therapy. The elevated right-hemispheric error rate in our study may be indicative of a partial shift of language function to the right hemisphere in nfvPPA patients during the course of disease and therefore might point to the existence of neuronal plasticity in neurodegenerative aphasia. Further, longitudinal studies are needed including larger patient samples and healthy controls to further investigate language function and brain plasticity in nvfPPA. In such larger cohort studies, our current data justifies investigating nrTMS error distribution as a new neurophysiological biomarker especially for the early phase of nfvPPA.

## Conclusions

Our pilot study showed that nrTMS is a feasible tool for language mapping in nfvPPA. Evaluating the individual language function might be a prerequisite for any targeted, personalized stimulation therapies. Study results suggests the existence of neuronal plasticity in neurodegenerative aphasia.

## Data Availability

The data that support the findings of this study are available from the corresponding author, upon reasonable request. The data are not publicly available due to ethical and legal restrictions. Due to German data protection rules transfer of data that is considered to be personal by local laws (for example video and sound recordings) is forbidden. For the same legal reasons transmission of data might be possible within the European Union only.
